# Case Report: Blepharophimosis and Ptosis as Leading Dysmorphic Features of Rare Congenital Malformation Syndrome With Developmental Delay – New Cases With *TRAF7* Variants

**DOI:** 10.3389/fmed.2021.708717

**Published:** 2021-08-26

**Authors:** Justyna Paprocka, Magdalena Nowak, Maria Nieć, Izabela Janik, Małgorzata Rydzanicz, Śmigiel Robert, Magdalena Klaniewska, Karolina Rutkowska, Rafał Płoski, Aleksandra Jezela-Stanek

**Affiliations:** ^1^Department of Pediatric Neurology, Faculty of Medical Sciences in Katowice, Medical University of Silesia, Katowice, Poland; ^2^Students' Scientific Society, Department of Pediatric Neurology, Faculty of Medical Sciences in Katowice, Medical University of Silesia, Katowice, Poland; ^3^Department of Medical Genetics, Medical University of Warsaw, Warsaw, Poland; ^4^Department of Paediatrics, Division of Propaedeutic of Paediatrics and Rare Disorders, Wroclaw Medical University, Wroclaw, Poland; ^5^Department of Genetics and Clinical Immunology, National Institute of Tuberculosis and Lung Diseases, Warsaw, Poland

**Keywords:** blepharophimosis, ptosis, developmental delay, *TRAF7* variants, facial features, dysmorphic features

## Abstract

Germline variants in tumor necrosis factor receptor-associated factor 7 (TRAF7) gene have recently been described in about 50 patients with developmental delay and cardiac, facial, and digital anomalies (CAFDADD). We aimed to depict further the clinical and genetic spectrum associated with *TRAF7* germline variants in two additional patients, broaden the mutational spectrum, and support the characteristic clinical variety to facilitate the diagnostics of the syndrome among physician involved in the evaluation of patients with developmental delay/congenital malformations.

## Introduction

*TRAF7* (Tumor necrosis factor receptor-associated factor 7) is a multifunctional intracellular protein belonging to the TRAF family (tumor necrosis factor receptor - TNF-R), which consists of seven proteins (TRAF1-7) (1, 2). It is encoded by a homonymous gene - *TRAF7* – encompassing 21 exons, located within the chromosomal region 16p13.3 (2). A modular structure characterizes all members of the TRAF family. TRAF7 670-amino acid protein contains an N-terminal RING finger domain (aa 125–160), a zinc finger domain (aa 221–287) and a coiled-coil domain, but instead of the classical TRAF domain, it has seven WD40 repeats at the C-terminus (1, 3).

TRAF proteins are active in many biological processes, including embryonic development, tissue homeostasis as well as innate and adaptive humoral immune responses (4, 5). *TRAF7* is involved in the process of protein ubiquitination. It is one of the critical mediators of NF-κB (nuclear factor-κB) and MAPK (mitogen-activated protein kinase) signaling pathways. It, therefore, plays an important role in many cellular processes (1–3, 6). The interaction of TRAF7 and MEKK3 (mitogen-activated protein kinase kinase kinase 3) through the WD40 domain leads to the activation of JNK and p38 MAP signaling pathways, which are responsible for cell survival, proliferation and differentiation (1–3). *TRAF7* also plays a specific role in muscle function. Reduced *TRAF7* expression under the influence of MyoD1 in growing myoblasts causes a decrease in NF-κB ubiquitination, exit from the cell cycle and acceleration of myogenesis (3, 6).

As a result of research conducted toward a closer understanding of TRAF7, a relationship between the protein and the development of neoplasms was demonstrated. Somatic mutations in *TRAF7* have been described in meningiomas, mesotheliomas, perineural tumors of soft tissues, as well as in glandular neoplasms of the genital tract (1, 7–9). In turn, germline mutations in this gene have been described in the neurodevelopmental syndrome, encompassing cardiac, facial, and digital anomalies with developmental delay, thus termed CAFDADD (OMIM #6181640), characterized by facial dysmorphism with specific anomalies within the eyes (ptosis, epicanthic folds), congenital defects of the heart (aortic coarctation, hypoplastic left heart, double outlet right ventricle) and skeletal system (distal contractures, overlapping digits), and psychomotor delay. The described to date germline variants are heterozygous missense mutations, including frequently repeated c.1964G>A (p. Arg655Gln) and occur *de novo* (2, 5, 10).

We present two patients with characteristic facial dysmorphic features in whom, as a result of performed Whole Exome Sequencing (WES), heterozygous *de novo* variants were detected in *TRAF7* gene: the c.1708C>G (p.His570Asp) in case 1 and c.1783C>G (p.Leu595Val) in case 2. Our aim is to delineate the phenotype and to underline the specific ocular findings that may facilitate the clinical diagnosis.

## Patients' Descriptions

### Case 1

A 2-month-old boy was admitted to the Department of Pediatric Neurology for the diagnosis of dysmorphic and ophthalmoplegic features. The boy was born in the 41st week of an uncomplicated pregnancy with the Apgar score of 10, bodyweight of 3,100 g and head circumference of 32 cm. The examination revealed a short two-vessel umbilical cord. He developed neonatal jaundice, which was treated with phototherapy. Transfontanelle ultrasound showed an asymmetric ventricular system. The patient's family history was unremarkable.

**Figure 1:**
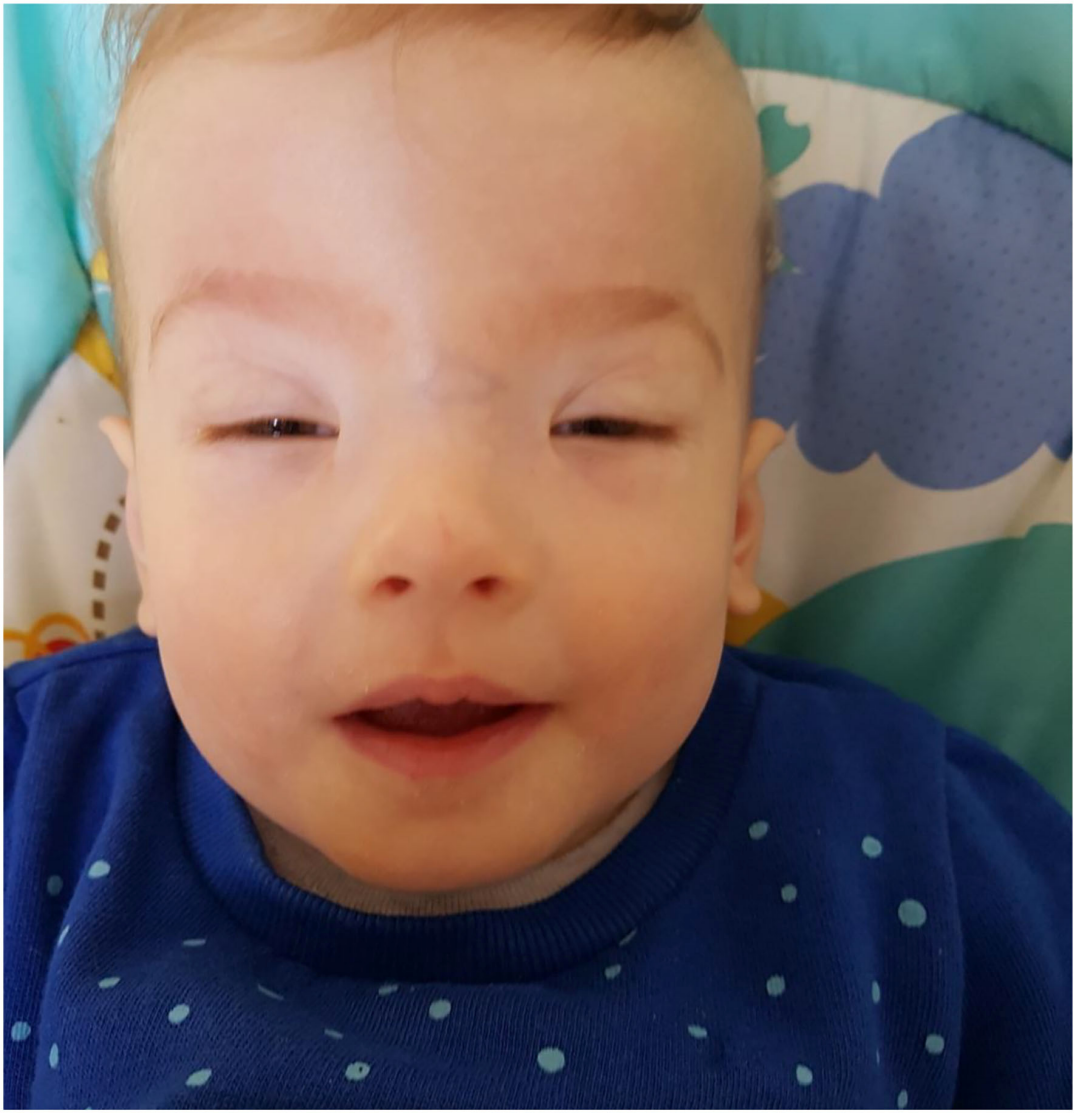
The facial phenotype of Case 1 at 11 months. Note the significant short palpebral fissures, ptosis, epicanthic folds and high forehead with flat, broad nasal bridge.

The child was diagnosed with facial dysmorphism, including swollen eyelids, small sunken eyes, which initially opened only 2–3 mm, as well as a wide nose with an upturned tip, a receding mandible and asymmetrical auricles; head circumference was 36 cm (<3rd percentile) and the frontal fontanel, sized 2 × 3 cm, was below the level of the skull bone. Moreover, the physical examination revealed a narrow chest with widely spaced nipples and significant binary stenosis (Figure 1).

Correct tendon-periosteal reflexes and symmetrical posture reflexes were found. Positional asymmetry with shortening of the left side and reduced muscle tension in the head-torso axis were revealed, and, for this reason, physical rehabilitation was recommended. Ophthalmoplegia was diagnosed by an ophthalmologist. The results of basic laboratory tests (morphology, urinalysis, capillary gasometry, liver function assessment, ionogram) were within the normal range.

MRI of the head and eye sockets was performed, which showed no abnormalities apart from the asymmetry of the bones of the skull cover. Suspecting the myogenic background of the described ptosis, the diagnostics was extended to include an electrostimulation test for measuring muscle fatigue, during which the right axillary nerve was stimulated with the registration of a response from the right deltoid muscle. No decrease in amplitude was found; therefore, the test result was determined as negative. In addition, the test for antibodies against acetylcholine receptors was performed, and the result was normal.

Echocardiography showed patent foramen ovale/atrial septal defect (PFO/ASD II) and patent ductus arteriosus (PDA) with a left-right shunt. The ultrasound of the abdomen and retroperitoneal space was also performed, which visualized the enlarged caliceal-pelvic system of the left kidney. *E. Coli* 10^4^ was obtained from the urine culture, and therefore a nephrological consultation was recommended. The patient was discharged with recommendations for further care in specialist units: cardiology, nephrology, ophthalmology, genetic counseling and physical rehabilitation clinics.

The patient was consulted by a geneticist, who recommended an array comparative genomic hybridization (aCGH) test. The result was normal. Thus, whole-exome sequencing (WES) was recommended for the suspected blepharophimosis, ptosis, epicanthus in vs. syndrome (BPES).

### Case 2

An almost 2-year-old boy is currently under the care of the Genetic Clinic because of a delay in psychomotor development and facial dysmorphic features. The boy was born by cesarean section at 40 weeks of gestation, with Apgar score of 8 points and birth weight of 4,120 g. The pregnancy was complicated by maternal infection and threatened preterm delivery. Family history was unremarkable.

After birth, no malformations of internal organs were found. Conductive hearing loss on the left side was identified. Due to clubfoot, the boy was referred to orthopedic consultation. The feet were successively plastered and splinted with good results. Cardiac ultrasound showed a bicuspid tricuspid valve. Ultrasound of the abdominal cavity showed a left-sided duplication of the caliceal-pelvic system. Cerebral NMR and angio-NMR showed hypoplasia of the middle and anterior segment of the sagittal sinus with peripheral venous circulation and slight dilatation of the supra-ventricular system as well as dilatation of brain fissures in the frontal and temporal regions and basal cisterns. TSH and CPK levels were normal. There were no abnormalities in the ophthalmological examination.

**Figure 2:**
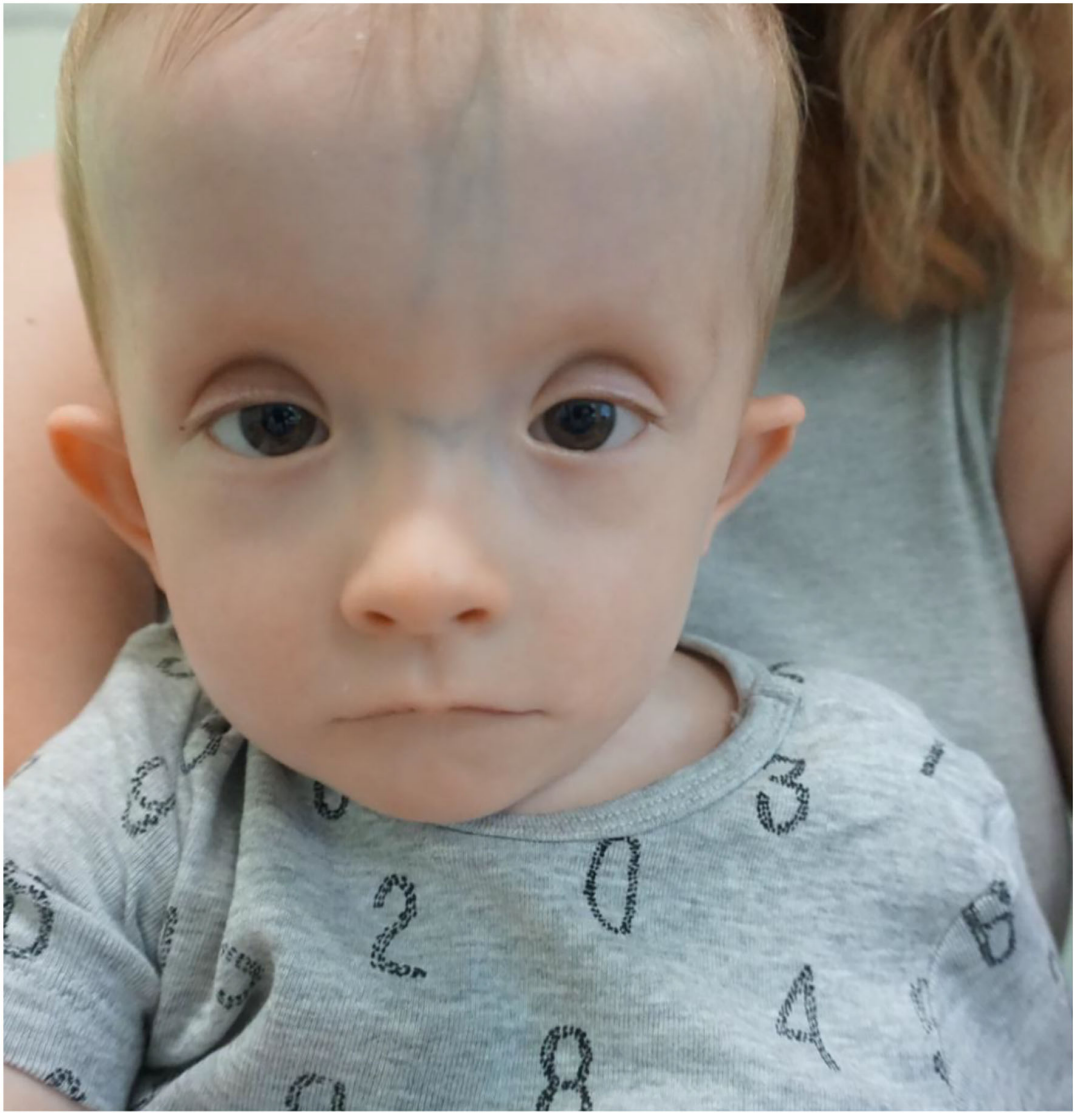
The facial phenotype of Case 2 and 16 months. Not the high and broad forehead, flat nasal bridge, short palpebral fissures and ptosis, thin lips.

Physical examination at the age of 16 months revealed specific features of craniofacial dysmorphia such as increased head circumference (OFC 50 cm – 97 percentile), prominent forehead and prominent veins on the forehead, two hair whirls as well as wide nasal bridge, short palpebral fissures, blepharophimosis and mild ptosis of both eyelids (Figure 2).

Palms and fingers were normal; however, the toes overlap. At 16 months of life, the weight was 10kg (25 percentile) and height 78 cm (25–50 percentile). Hypotonia was also found. The boy is intensively rehabilitated. He sat up by himself at the age of 12 months and started walking at the age of 2 years. He does not speak; however, according to his parents, he understands speech.

Cytogenetic examination showed normal male karyotype. Array CGH did not reveal any chromosomal aberrations. Clinically dysmorphic syndrome belonging to the RASopathies group was suspected in the child. Therefore, a whole-exome sequencing (WES) was performed.

For both probands, WES analysis was performed using SureSelectXT Human kit All Exon v7 - Case 1 and v5 – Case 2 (Agilent, Agilent Technologies, Santa Clara, CA). Enriched libraries were paired-end sequenced (2 × 100 bp) on HiSeq 1,500 (Illumina, San Diego, CA, USA) and analyzed as previously described (11). In brief, raw sequence readouts were initially analyzed with *bcl2fastq* software (Illumina) to generate reads in fastq format. After the quality control steps, including adapter trimming and low-quality read removal, reads were aligned to the GRCh38 (hg38) reference genome with Burrows-Wheeler Alignment Tool (http://bio-bwa.sourceforge.net/), and processed further by Picard (http://broadinstitute.github.io/picard/) and Genome Analysis Toolkit (https://software.broadinstitute.org/gatk/). Identified variants were annotated with functional information, frequency in population (including gnomAD (http://gnomad.broadinstitute.org/), and an in-house database of >3,500 Polish exomes), and known association with clinical phenotypes, based on both ClinVar (https://www.ncbi.nlm.nih.gov/clinvar/) and HGMD (http://www.hgmd.cf.ac.uk) databases. *In silico* pathogenicity prediction was performed based on Varsome pathogenicity and conservation scores (12) and by MetaSVM (13).

Variants passing a default quality were further filtered to include only those with <1% minor allele frequency in all tested databases (gnomAD, in house database of >3,500 Polish exomes), and to exclude deep intronic variants. The final list of variants were screened against known pathogenic mutations listed in ClinVar an dHGMD databases, and then searched for biallelic mutations consistent with autosomal recessive inheritance and monoallelic variants potentially causative of an autosomal dominant. All prioritized variants were manually inspected with Integrative Genomics Viewer (14).

Enriched libraries were paired-end sequenced (2 × 100 bp) on HiSeq 1,500 (Illumina, San Diego, CA, USA). Raw sequencing data and variants prioritization were performed as previously described (11). For variants considered as disease-causing segregation analysis in probands' families was performed by amplicon deep sequencing (ADS) performed by Nextera XT Kit (Illumina) and sequenced as described for WES.

**Figure 3:**
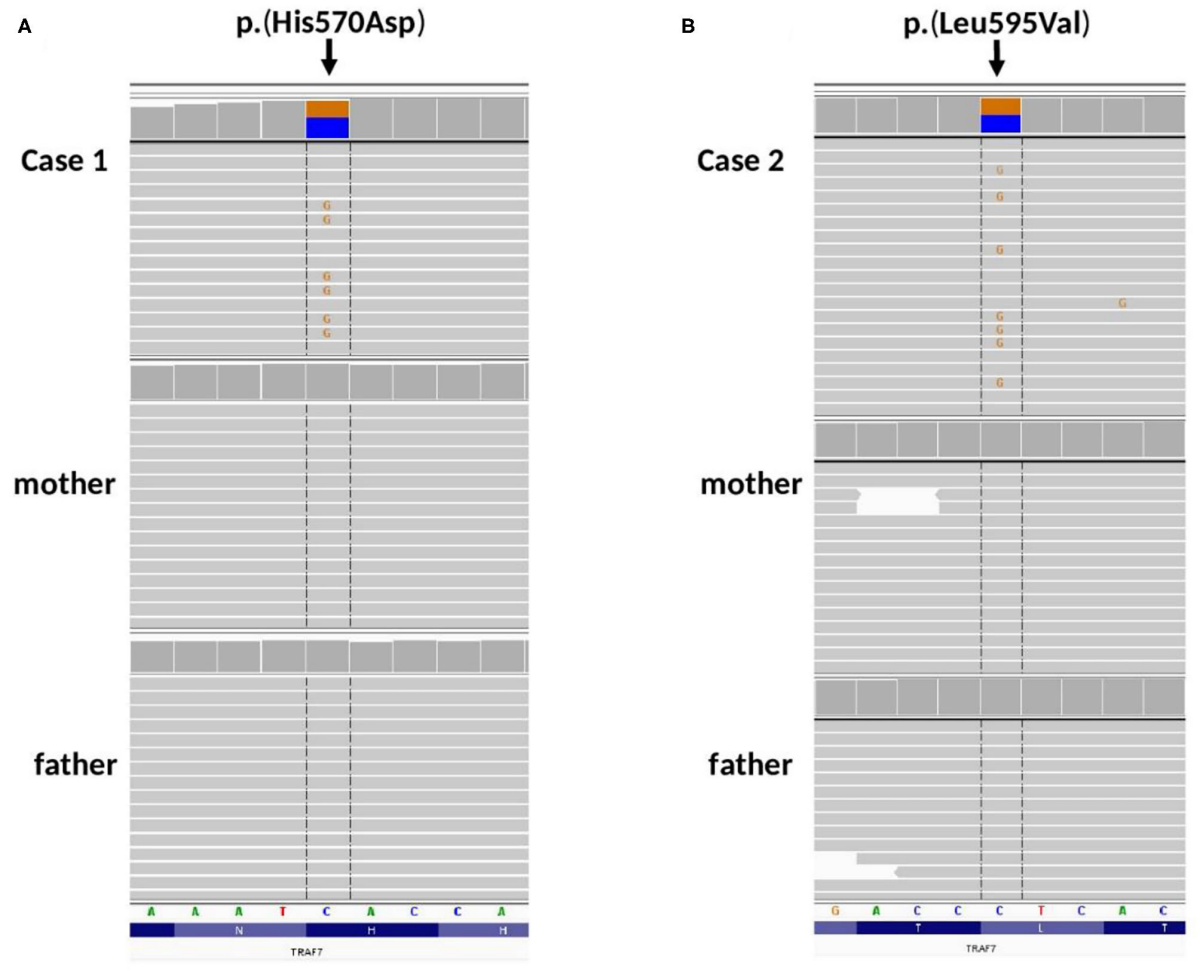
Amplicon deep sequencing validation of TRAF7 gene variants identified by WES in **(A)** Case 1, *de novo* p.(His570Asp), **(B)** Case 2, *de novo* p.(Leu595Val). Integrative Genomic Viewer screenshots are presented.

In Case 1, ultra-rare variants in *LEMD3, RAI1* and *TRAF7* genes were prioritized for further validation. Variants in *LEMD3, RAI1* were found to be inherited from proband's healthy mother and were classified as benign (data not shown). Whereas, missense heterozygous variant in *TRAF7* gene [(hg38, chr16:g.002175915-C>G, NM_032271.3: c.1708C>G, p.(His570Asp)] was absent in both parents (Figure 3A) and considered as *de novo* event.

In Case 2, ultra-rare variants in *CHD1, NAV2, KIF3B* and *TRAF7* genes were prioritized for segregation study. Variants in *CHD1, NAV2, KIF3B* were inherited from healthy parents (*CHD1* from a father, *NAV2, KIF3B* from a mother, date not shown) and were considered as benign. Missense heterozygous variant in *TRAF7* gene [(hg38, chr16:g.002176085-C>G, NM_032271.3: c.1783C>G, p.(His595Asp)] was absent in both parents (Figure 3B) and considered as *de novo* event.

Both p.(His570Asp) and p.(Leu595Val) have 0 frequency in all tested databases (including the in-house database of >4,500 Polish individuals examined by WES). According to ACMG classification (15) p.(His570Asp) variant is classified as “Likely Pathogenic,” while p.(Leu595Val) as “Variant of Unknown Significance.” Additionally, c.1783C>G (p.Leu595Val) was predicted as damaging/pathogenic by 16 out of 21 pathogenicity predictors implemented by Varsome (including MutationTaster, SIFT, FATHMM), and as damaging by MetaSVM.

## Discussion

Germline mutations in *TRAF7* and the associated clinical symptoms were first described in 2018 by Tokita et al. *De novo* missense mutations in *TRAF7* were found in 7 patients with diagnosed developmental delay, congenital defects and dysmorphic features (2). Castilla-Vallmanya et al., on the basis of the identification of 45 patients, defined this disease entity as *TRAF7* syndrome characterized by specific features of facial dysmorphia, especially eyelid fissure defects, congenital heart and skeletal defects, as well as motor retardation and intellectual disability (10). To date, 54 cases of patients with a germline mutation in *TRAF7* have been described in the literature (2, 5, 10).

*De novo* variant p.(His570Asp) identified in Case 1 was previously reported in two independent CAFDADD patients (10). While p.(Leu595Val) identified in Case 2 has not been described in the literature so far.

We present a case of a patient with a *de novo* heterozygous *TRAF7* mutation. The pathogenic nature of this variant and its participation in the clinical picture of the patient is indicated by the characteristic clinical symptoms consistent with the phenotypes of patients with diagnosed *TRAF7* germinal mutations (2, 10), the applied bioinformatics programs and the exclusion of its occurrence in both parents.

The phenotype revealed in the patient, and primarily the occurrence of blepharophimosis and epicanthic folds, highlight the intensity of dysmorphic features within the eyes, which is characteristic of the CAFDADD syndrome. In addition, the described features such as broad nasal base, short neck, dysmorphic, asymmetrically placed auricles, and a retracted mandible (retrognathia) complete the picture of typical phenotype features characteristic of *TRAF7* germline mutations (2, 10). The skeletal defects, manifested by significant bilateral narrowing of the skull, raise the suspicion of craniosynostosis, which is one of the most frequently described skull defects in patients with *TRAF7* mutations (5, 10). Therefore, the CAFDADD syndrome should be included in the spectrum of genetic defect syndromes, in which the neurosurgical assessment of the patient and constant monitoring of their condition and development is of key importance in order to avoid possible complications of craniosynostosis through properly implemented treatment (16–18). The patent ductus arteriosus and patent foramen ovale diagnosed in the patient are typical cardiac manifestations of the CAFDADD syndrome, which indicates the need for constant cardiological supervision (2, 10). Due to the previously described developmental disorders such as psychomotor retardation, intellectual disability and speech disorders, the support of early child development as well as physical rehabilitation, aimed at compensating for frequently occurring muscle tension disorders, is of key importance (2, 5, 10).

Primarily, the CAFDADD syndrome phenotype includes distinctive dysmorphic features in the eye area, which can often be the first abnormalities noticed. Thus, it is of importance for child ophthalmologists to keep the syndrome in mind during the evaluation of the patients with such features having other developmental anomalies (physical as well as delayed psychomotor milestones). The patient's disorders of eye-opening indicated a clinical picture characteristic of ophthalmic forms of myasthenia gravis (19). The exclusion of myasthenia gravis by means of a negative result of the electrostimulation-induced muscle fatigue test led to further diagnostics toward a genetic syndrome with severe ptosis, narrow eyelid cracks and epicanthic folds.

Initially, the blepharophimosis-ptosis-epicanthus invertus syndrome (BPES) was suspected in the presented Case 1. BPES is a complex eyelid defect syndrome characterized by four main features: blepharophimosis, eyelid ptosis, epicanthus invertus and telecanthus (BPES II), as well as premature ovarian failure in BPES I (20). In both BPES and CAFDADD, a palpebral fissure defect is one of the characteristic clinical features. If BPES is suspected, it is necessary to exclude CAFDADD, in which there are both systemic complications, especially the heart and skeletal defects, as well as possible predisposition to cancer development and premature aging, absent in BPES.

In patients with a suspected *TRAF7* mutation in the differential diagnosis, Ohdo's syndrome, which is characterized by blepharophimosis, ptosis, congenital disorders of the heart and limbs, and developmental delay, should also be considered (21, 22). In previously described patients, RASopathy group diseases were also suspected, e.g., Noonan syndrome and Costello syndrome (2, 5, 10). Of note, the RASopathy was initially suspected in presented Case 2. The common pathomechanism of RASopathy associated with dysregulation of the Ras/MAPK signaling pathways causes a characteristic clinical picture: features of craniofacial dysmorphia, heart defects, ocular and musculoskeletal disorders (23). The overlapping phenotypes of RASopathy and the CAFDADD syndrome and the suspected involvement of *TRAF7* in the regulation of MAPK signaling pathways suggest a possible correlation in the pathomechanisms (1, 2, 5).

Somatic mutations in *TRAF7* have been detected in such neoplasms as meningiomas, mesotheliomas, perineural tumors of soft tissues or glandular neoplasms of the genital tract (1, 23). Most of them are missense mutations located within the WD40 domain. The germline mutations in *TRAF7* are also mostly clustered within the WD40 domain (1, 10). For this reason, constant supervision in aging patients is required to determine whether they have a higher risk of cancer development and should be included in the oncological surveillance (10).

## Conclusions

The detected *de novo* variant c.1708C>G [p.(His570Asp)] in *TRAF7* and the described phenotype correlating with it allow to extend of the genetic spectrum of the very rare CAFDADD syndrome (Cardiac, facial and digital anomalies with developmental delay). Moreover, the presented known variant - c.1783C>G, p.(His595Asp) – support the previous findings concerning the phenotypic spectrum of *TRF7* germline variants. A *TRAF7* mutation should be suspected in patients with characteristic dysmorphic features, especially within the palpebral fissure (blepharophimosis and/or ptosis), congenital defects of the heart and skeleton, and psychomotor delay. As we proved herein, the clinical manifestation may differ and overlaps with other, more frequent genetic condition as Noonan syndrome (RASopathies). Constant ophthalmic, neurological and cardiological assessment, as well as early development support and motor rehabilitation, are essential in the management of patients with the syndrome resulting from variants in *TRAF7*.

## Data Availability Statement

The original contributions presented in the study are included in the article/supplementary materials, further inquiries can be directed to the corresponding author/s.

## Ethics Statement

Written informed consent was obtained from the parents for the publication of any potentially identifiable images or data included in this article.

## Author Contributions

Conceptualization was performed by JP and AJ-S. Methodology, formal analysis, investigation, resources, data curation, and project administration were performed by JP, MNo, MNi, IJ, MR, ŚR, RP, and AJ-S. Software by validation was performed by JP, MNo, MNi, IJ, and AJ-S. Writing-original draft preparation, writing-review and editing, and visualization were performed by JP, MNo, MNi, IJ, MR, ŚR, MK, KR, RP, and AJ-S. Supervision was performed by AJ-S. Funding acquisition was performed by JP. All authors have read and agreed to the published version of the manuscript.

## Conflict of Interest

The authors declare that the research was conducted in the absence of any commercial or financial relationships that could be construed as a potential conflict of interest.

## Publisher's Note

All claims expressed in this article are solely those of the authors and do not necessarily represent those of their affiliated organizations, or those of the publisher, the editors and the reviewers. Any product that may be evaluated in this article, or claim that may be made by its manufacturer, is not guaranteed or endorsed by the publisher.
